# Redox Signaling Is an Early Event in the Pathogenesis of Renovascular Hypertension

**DOI:** 10.3390/ijms140918640

**Published:** 2013-09-10

**Authors:** Stella P. Hartono, Bruce E. Knudsen, Adeel S. Zubair, Karl A. Nath, Stephen J. Textor, Lilach O. Lerman, Joseph P. Grande

**Affiliations:** 1 Mayo Medical School, Mayo Clinic, 200 First Street SW, Rochester, MN 55905, USA; E-Mails: hartono.stella@mayo.edu (S.P.H.); zubair.adeel@mayo.edu (A.S.Z.); 2 Medical Scientist Training Program, Mayo Clinic, 200 First Street SW, Rochester, MN 55905, USA; 3 Department of Laboratory Medicine & Pathology, Mayo Clinic, 200 First Street SW, Rochester, MN 55905, USA; E-Mail: knudsen.bruce@mayo.edu; 4 Division of Nephrology & Hypertension, Mayo Clinic, 200 First Street SW, Rochester, MN 55905, USA; E-Mails: nath.karl@mayo.edu (K.A.N.); textor.stephen@mayo.edu (S.J.T.); lerman.lilach@mayo.edu (L.O.L.)

**Keywords:** renovascular hypertension, renin-angiotensin-aldosterone system, oxidative stress, inflammation

## Abstract

Activation of the renin-angiotensin-aldosterone system plays a critical role in the development of chronic renal damage in patients with renovascular hypertension. Although angiotensin II (Ang II) promotes oxidative stress, inflammation, and fibrosis, it is not known how these pathways intersect to produce chronic renal damage. We tested the hypothesis that renal parenchymal cells are subjected to oxidant stress early in the development of RVH and produce signals that promote influx of inflammatory cells, which may then propagate chronic renal injury. We established a reproducible murine model of RVH by placing a tetrafluoroethhylene cuff on the right renal artery. Three days after cuff placement, renal tissue demonstrates no histologic abnormalities despite up regulation of both pro- and anti-oxidant genes. Mild renal atrophy was observed after seven days and was associated with induction of *Tnfα* and influx of CD3+ T cells and F4/80+ macrophages. By 28 days, kidneys developed severe renal atrophy with interstitial inflammation and fibrosis, despite normalization of plasma renin activity. Based on these considerations, we propose that renal parenchymal cells initiate a progressive cascade of events leading to oxidative stress, interstitial inflammation, renal fibrosis, and atrophy.

## 1. Introduction

Renal artery stenosis (RAS) is a common public health problem, being present in almost 7% of individuals older than 65 years of age [1]. Patients with RAS often have generalized atherosclerosis [2]. In addition to secondary hypertension and development of chronic renal failure, patients with RAS are at high risk for cardiovascular death [3]. In a recent study, Conlon *et al.* reported that the 4-year survival of patients undergoing cardiac catheterization was 86% in patients without RAS but only 65% in those with RAS [4]. The extent of RAS also predicts survival, with 4-year survival of 89% in patients with RAS <75% luminal occlusion, but only 57% in those with >75% luminal occlusion [5].

The optimal management of patients with RAS is not clear. Several studies have indicated that improvement in blood pressure control and recovery of renal function is achieved in less than half of patients undergoing renal revascularization [6–8]. The recent Angioplasty and Stenting for Renal Artery Lesion (ASTRAL) Study demonstrated that percutaneous transluminal renal angioplasty (PTRA) fails to improve renal function, blood pressure, renal or cardiovascular events, or mortality [9]. From these studies, it is clear that other factors in addition to renal perfusion affect outcome in patients with RAS. Therefore, better understanding of the sequence of events that lead to irreversible renal damage may provide the basis for novel therapeutic targets.

While the role of Ang II in the development of oxidative stress and of chronic renal disease in RAS is well established, it is not clear how the multiple pathways triggered by Ang II intersect to produce chronic interstitial inflammation, interstitial fibrosis, and tubular atrophy, the morphologic hallmarks of irreversible renal injury. In particular, it is not known whether interstitial inflammation is a cause or a consequence of oxidative stress. Previous studies have focused more on the role oxidative stress once chronic renal injury has been established. The objective of this study was to study early time points in the development of renal damage to define the relationship between oxidative stress and interstitial inflammation. We sought to test the hypothesis that oxidative stress occurs prior to the influx of inflammatory cells, and mediated by renal parenchymal cells.

## 2. Results

### 2.1. RAS Mice Rapidly Develop Hypertension and Cardiac Hypertrophy

**Figure 1:**
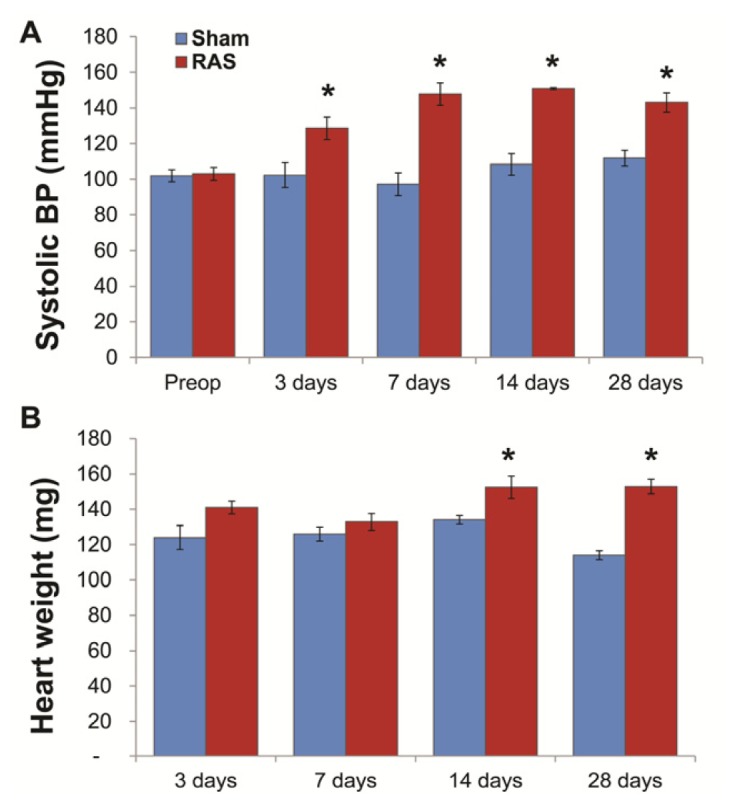
(**A**) Systolic blood pressure (BP) in mice with renal artery stenosis (RAS) (*N* = 10) was higher compared to mice with sham surgery (*N* = 5) at all time-points. BP was measured in conscious mice pre-surgery and at 3, 7, 14, and 28 days post surgery; (**B**) Mean heart weight in mice with RAS was significantly increased at 14 days following RAS surgery compared to mice with sham surgery. *p < 0.05 in comparison to sham.

In our previous studies, we have characterized the structural and functional alterations in the murine RAS model beginning at two weeks after surgery. In this study, we have expanded our studies to include alterations observed at early time points—three and seven days following RAS surgery. Mice with RAS develop hypertension within three days of surgery, from a baseline of 102 ± 2 mmHg to 129 ± 6 mmHg (*p* < 0.05). As observed in our previous studies with this model [10–12], blood pressure in RAS mice remained elevated throughout the 28 days observation period (Figure 1A). Mean heart weight was significantly increased at 14 days following RAS surgery and remained elevated thereafter (Figure 1B) [10–12].

### 2.2. Renal Atrophy Begins to Develop at Three Days Following RAS Surgery

**Figure 2:**
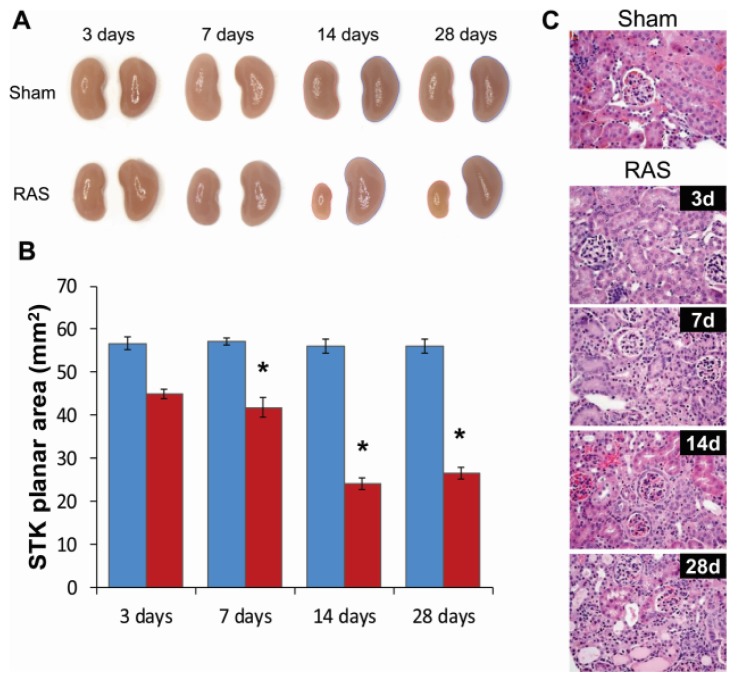
(**A**) Representative gross images of the kidneys from mice with RAS and sham surgery at 3, 7, 14, and 28 days; (**B**) Planar surface measurements of the stenotic kidney (STK) of mice with RAS (*N* = 10) and sham (*N* = 5) surgeries; (**C**) Representative histological images of stenotic kidney of mice with RAS surgery at 3, 7, 14, and 28 days post-surgery and mice with sham surgery at three days post-surgery as stained with hematoxylin and eosin (H & E). All images were taken at 400×. *p < 0.05 in comparison to sham.

**Figure 3:**
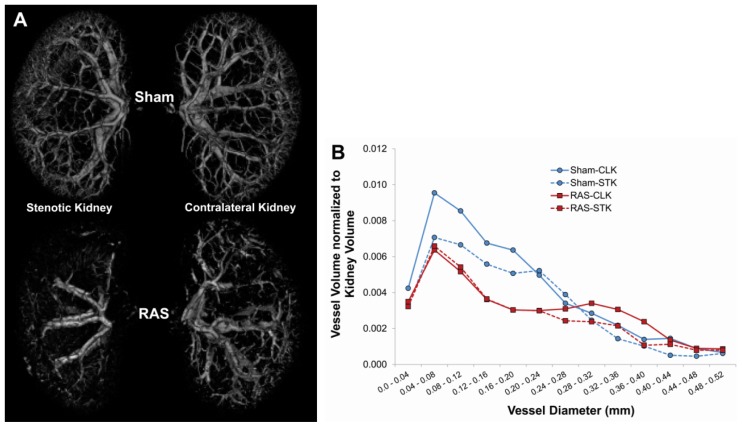
(**A**) Representative micro CT images of kidneys from mice with sham (*N* = 3) and RAS (*N* = 3) surgery, showing loss of vascular density in both stenotic and contralateral kidneys; (**B**) Both stenotic (STK) and contralateral (CLK) kidneys of mice subjected to RAS surgery showed loss of microvessel density. Representative figure showed representative distribution of vessels with diameter <0.52 mm in kidneys of mice with sham and RAS surgery. Vessel distribution was calculated as ratio of vessel volume to total kidney volume.

We measured planar surface area of the kidneys to determine the extent of organ atrophy. We found that the cuffed kidney showed a modest decrease in size during the first week and, in accord with our previous studies [10–12], developed severe atrophy after two weeks (Figure 2A,B). At three days following RAS, the cuffed kidneys showed minimal histopathologic alterations (Figure 2C). There was no significant interstitial fibrosis or tubular atrophy, or evidence of acute kidney injury, including tubular epithelial cell necrosis, capillary dilation, or neutrophil infiltrates. At seven days following RAS surgery, the renal cortex showed diffuse mild atrophy, characterized by mild flattening and simplification of tubular epithelium. By 28 days following RAS surgery, the cuffed kidney showed generalized tubular atrophy, as evidenced by flattening and simplification of the proximal tubular epithelial cells and focal thickening of the tubular basement membranes. Micro CT analysis demonstrated a 17.5% loss of microvessel density in the cuffed kidney of RAS mice as compared to the sham mice after four weeks (Figure 3A,B).

**Figure 4:**
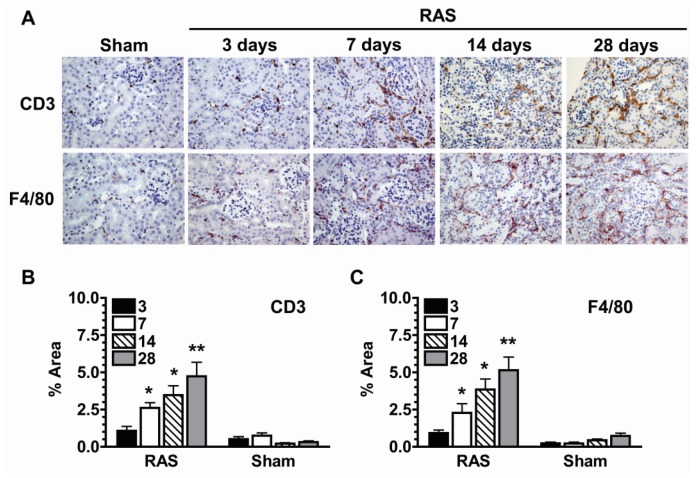
(**A**) Representative histological images of stenotic kidney of mice with RAS (*N* = 10) surgery at 3,7,14, and 28 days post-surgery and mice with sham (*N* = 5) surgery at three days post-surgery as stained with anti-CD3 (T-cells) and anti-F4/80 (macrophages) antibody. All images were taken at 400×; (**B**) Extent of T-cell infiltration as assessed by quantifying % area positive for CD3 stains; (**C**) Extent of macrophage infiltration as assessed by quantifying % area positive for F4/80 stains. Assessments were done at 200× magnification over the whole cortex using color assisted image analysis. *p < 0.05, **p < 0.01 in comparison to sham.

Histologic evaluation of H&E stained sections did no show appreciable increase in neutrophil or mononuclear leukocyte influx at three days (Figure 2C). To determine the extent of inflammation in the stenotic kidney, we performed immunostains for infiltrating CD3+ T cells and F4/80+ macrophages. There were no significant increases in the number of interstitial CD3+ T cells and F4/80+ macrophages in the first week following RAS (Figure 4A–C). The number of infiltrating cells increased after one week and paralleled the development of renal atrophy at subsequent time points [10–12].

### 2.3. RAS Is Associated with Early Production of Superoxide Anion

**Figure 5:**
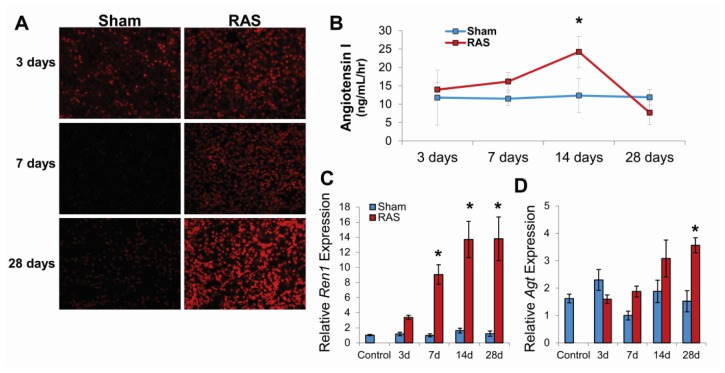
(**A**) Mice with sham (*N* = 5) surgery experienced oxidative stress (DHE stains) only at three days post-surgery while mice with RAS (*N* = 10) surgery experienced increasing level of oxidative stress; (**B**) Plasma renin activity (as assessed by angiotensin I production) is elevated by three days post-surgery in mice with RAS and peak at 14 days post-surgery before returning to baseline level by 28 days post-surgery; (**C**) Renin (*Ren1*) expression was up regulated in the stenotic kidney of mice with RAS at all time-points; (**D**) Angiotensinogen (*Agt*) expression in the stenotic kidney of mice with RAS became significantly elevated at 28 days post-surgery. For (**C**) and (**D**), data was normalized against 18s transcript and expressed as relative expression compared to the seven day sham. *p < 0.05 in comparison to sham.

Representative photomicrographs of kidneys obtained from RAS and sham mice at 3, 7, and 28 days following surgery are shown in Figure 5A. After three days, over 80% of tubular epithelial cell nuclei showed strong positive staining with DHE. After seven and 28 days, the proportion of cells staining positively for DHE was similar, but the intensity of staining increased. In contrast, the 3-day sham control showed positive staining in approximately 40% of cells (presumably reflecting the effects of surgical manipulation of the vascular pedicle); the seven and 28-day sham controls showed minimal staining.

Plasma renin activity, as assessed by angiotensin I content began to increase within three days of RAS surgery; this increase in level achieved statistical significance at 14 days (Figure 5B). In accordance with our previous observations, plasma renin activity returned to baseline levels at 28 days following surgery [10,12]. To determine the extent of intra-renal activation of renin-angiotensin system, we measure the level of renin and angiotensinogen production in the kidney cortex by RT-PCR. RAS mice showed up regulation of Ren1 by three days post-surgery that became significant at seven days, peaked at 14 days, and sustained at the same level at 28 days (Figure 5C). On the other hand, angiotensinogen was not significantly up regulated until 28 days post-surgery (Figure 5D), which suggest that despite the decrease in plasma renin activity there was intra-renal activation of renin-angiotensin system within the stenotic kidney.

### 2.4. RAS Is Associated with Early Expression of Oxidant-Related Genes

**Figure 6:**
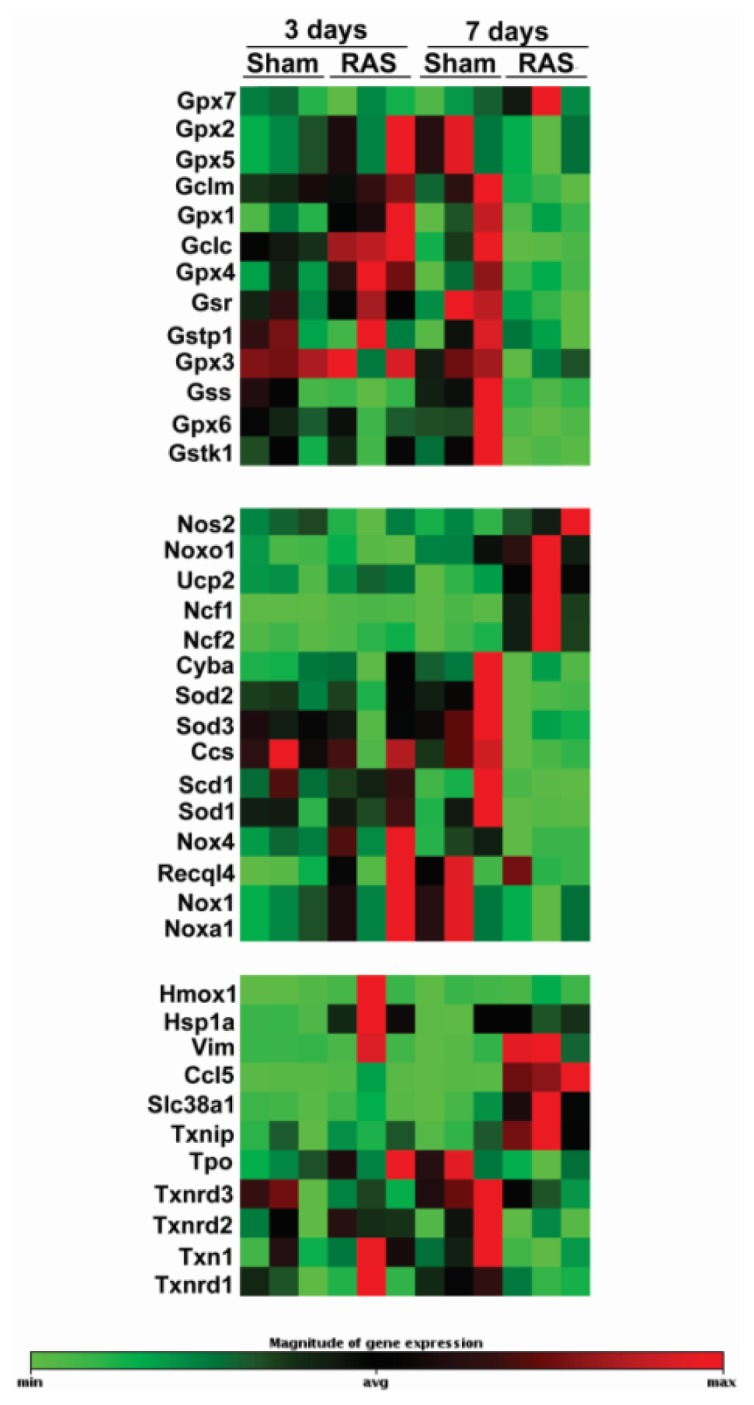
The clusterograms (heat maps) represent relative expression levels for all samples on genes showing significant changes on Oxidative Stress and Antioxidant Defense PCR Array from SABiosciences. *N* = 3 for each group.

In order to study early events in the development of RAS, we performed oxidative pathway analysis on renal cortex isolated at three and seven days following RAS surgery. A summary of expression patterns is shown in Figure 6. At three days, significant induction of *Gpx1*, *Gpx4*, *Hspa1a*, and *Ncf1* was observed. No significant down regulation of oxidant-related genes was observed. At seven days, at which time mild tubular atrophy was first observed, significantly up regulated genes included *Ncf1*, *Ucp2*, *Vim*, *Txnip*, and *Ccl5*. We also observed significant down regulation of anti-oxidant genes at this time point, including *Cat*, *Ccs*, *Fmo2*, *Gpx3*, *Sod2*, *Sod3*, *Prdx3*, and *Prdx5*.

**Figure 7:**
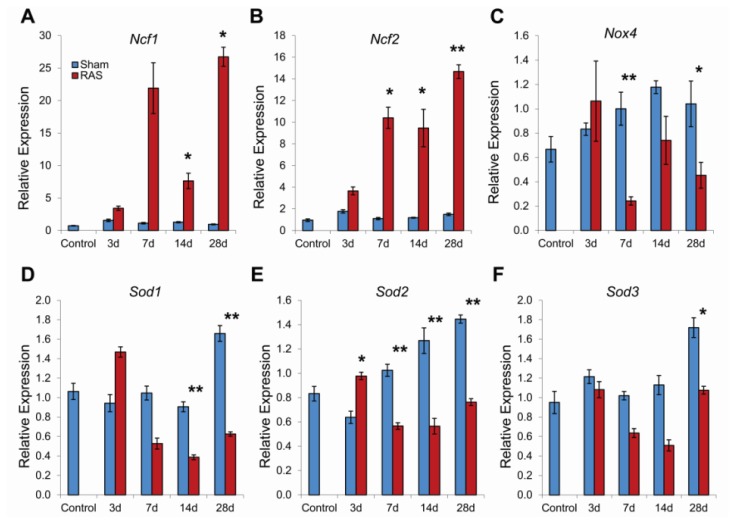
Kidney gene expression changes in *Ncf1* (**A**), *Ncf2* (**B**), *Nox4* (**C**), *Sod1* (**D**), *Sod2* (**E**), and *Sod3* (**F**) associated with renal artery stenosis (RAS). Data (means ± SE) for each gene was normalized to 18s transcripts and expressed as relative expression compared to the seven day sham. *N* = 10 for RAS groups at each time points. *N* = 5 for sham groups at each time points. Control group (*N* = 5) refers to mice without surgery. *p < 0.05, **p < 0.01 in comparison to sham.

To determine if this pattern of up regulation of oxidant-related genes along with down regulation of anti-oxidative genes is sustained throughout RAS progression, we performed RT-PCR validation of *Ncf1*, *Ncf2*, *Nox4*, *Sod1*, *Sod2*, *Sod3*. Both *Ncf1* and *Ncf2* showed a 2-fold increase at three days that developed to over 10 fold increase by seven days and sustained at that level up until 28 days (Figure 7A,B). However, *Nox4* expression in RAS mice was consistently down regulated (Figure 7C). *Sod1* and *Sod2* showed a slight increase at three days, but all of the SOD isoforms showed a significant decrease compare to sham at 7, 14 and 28 days (Figure 7D–F).

### 2.5. Parenchymal Cells Initiate Early Oxidative Stress Signaling

To further define the contribution of parenchymal versus infiltrating inflammatory cells at early time points following RAS surgery, we isolated CD90+ T cells and CD11b+ macrophages from renal cortex by magnetic separation and compared gene expression by RT-PCR with the CD11b−/CD90− fraction, which was enriched in parenchymal cells.

**Figure 8:**
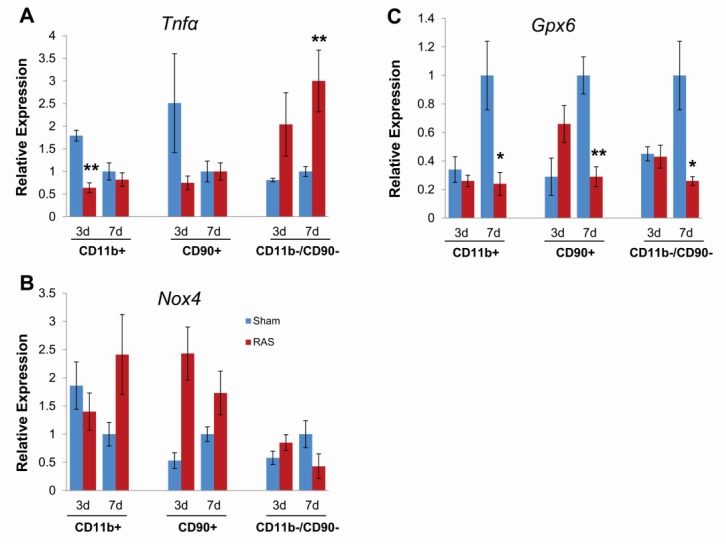
Gene expression changes in *Tnfα* (**A**), *Nox4* (**B**), and *Gpx6* (**C**) on CD11b+ (macrophages), CD90+ (T-cells), and CD11b−/CD90− (kidney parenchymal cells) fractions. Data (means ± SE) for each gene was normalized to 18s transcripts and expressed as relative expression compared to the seven day sham. *N* = 5 for both RAS and sham groups at each time points. *p < 0.05, **p < 0.01 in comparison to sham.

At both three and seven days, we observed a significant induction of TNFα expression in the CD11b−/CD90− (parenchymal cell) fraction isolated from RAS mice as compared to their sham control, but no significant induction of *TNFα* expression in the CD90+ (T cell) or CD11b+ (macrophage) fractions (Figure 8A). The NADPH oxidase subunit *Nox4* was induced in T cells, but not macrophages or parenchymal cells at three days. At seven days, *Nox4* was induced in macrophages and T cells, but was decreased in parenchymal cells (Figure 8B). These findings indicate that the reduction in *Nox4* expression observed in renal cortex (Figure 7C) reflects decreased expression by parenchymal cells. The antioxidant gene *Gpx6* was induced in T cells but not parenchymal cells or macrophages at three days and was down regulated in all three fractions at seven days (Figure 8C). Based on these considerations, we conclude that early oxidative stress signals following RAS arise from parenchymal cells rather than infiltrating inflammatory cells.

## 3. Discussion

Optimal management of patients with RAS is a vexing clinical issue, as these patients are at significantly increased risk for morbidity and mortality due to cardiovascular and cerebrovascular diseases in addition to chronic renal disease [13]. There is increasing evidence that reperfusion of the stenotic kidney fails to improve renal outcome in many cases [9]. In a porcine model of RAS, percutaneous transluminal angioplasty improved glomerular filtration rate and renal endothelial function, but failed to fully reverse the changes in microvascular structure that are associated with development of chronic renal disease in this model [14]. In addition to loss of microvascular density, renal inflammation and oxidative stress persisted after revascularization [15]. Furthermore, progression of renal disease was observed despite normalization of plasma renin activity [16,17]. Relatively few studies have focused on early events in the development of renal atrophy, before the onset of irreversible injury and presumably during a time where restoration of blood flow may have therapeutic benefit.

To address this deficiency, we have extended our previous studies of signaling pathways triggered by RAS [10–12] to include earlier events (three and seven days following surgery) and to survey, for the first time, oxidant responsive genes that are expressed following induction of RAS. We have previously established that placement of a cuff on the right kidney produces a reproducible decrease in blood flow (approximately 70%) that is stable from three days to at least four weeks after surgery [10]. This reproducible model allows us to critically evaluate both early signaling events that ultimately lead to the development of systemic hypertension and of renal atrophy in the cuffed kidney.

In this study, we provide a more comprehensive picture of the renin-angiotensin system at early time points following RAS surgery. Mice became hypertensive within three days of RAS surgery, although plasma renin activity was not yet significantly increased. At this early time point, renal expression of *Ren1* was significantly induced. Several studies have emphasized an important role for local autocrine or paracrine activation of the renin angiotensin system in regulation of blood pressure [18,19]. It is known that the kidney expresses all elements of the renin angiotensin system: renin, angiotensinogen, angiotensin converting enzyme, and angiotensin type 1 and type 2 receptors [18,20]. In the kidney, renin is expressed primarily in the juxtaglomerular cells. Angiotensinogen is expressed in proximal tubular epithelial cells and is secreted into tubular lumens. Angiotensin I is converted to Angiotensin II through action of ACE located on the apical brush border of tubular epithelium [20]. Based on these considerations, we propose that intra-renal activation of the renin-angiotensin system plays an important role in the development of renovascular hypertension.

It is likely that intra-renal activation of the renin-angiotensin system also plays an important role in maintenance of blood pressure at later time points, during the development and progression of chronic renal damage. In accord with our previous observations [10,12,16,17], we found that plasma renin activity was transiently elevated in mice subjected to RAS, peaking at 14 days after surgery and returning to baseline levels by 28 days. This normalization of plasma renin content was associated with the development of severe atrophy in the cuffed kidney. On the other hand, renal expression of *Ren1* and angiotensinogen progressively increased with time; highest inductions were seen at later time points when plasma renin activity had returned to baseline levels. Based on these considerations, we propose that intra-renal activation of the renin angiotensin system may be responsible for the continued elevation of blood pressure and production of reactive oxygen species, as evidenced by strong DHE staining at later time points, when the stenotic kidney has developed interstitial fibrosis and tubular atrophy, with interstitial inflammation.

In this study, we provide, for the first time, an analysis of the early renal morphologic changes associated with the development of renovascular hypertension in RAS. Histopathologic analysis of renal tissue at three days after surgery revealed no significant abnormalities in either cortex or medullary regions, despite a modest reduction in kidney size, as assessed by planar surface area. Of note, there was no evidence of acute kidney injury (acute tubular necrosis). Stains for CD3+ T cells and F4/80+ macrophages did not reveal any significant infiltration, compared to sham age matched controls. Furthermore, our PCR array study showed that *Ccl5* (*RANTES*) was not elevated yet at three days. The lack of significant inflammation at this time point indicates that the early signals that promote chronic renal damage may come from parenchymal cells. To provide further support for this hypothesis, we showed that the strongest induction of TNFα mRNA expression was from the CD90−, CD11b− (parenchymal cell) fraction, rather than from infiltrating T cells or macrophages at this early time point. At later time points, in accordance with our earlier studies [10–12], we observed progressive renal atrophy, which was associated with influx of T cells and macrophages, interstitial fibrosis, and loss of micro vessel density.

It is well established that, in RAS, Ang II promotes chronic renal injury through induction of reactive oxygen species generation [16,21,22]. Ang II promotes ROS generation through activation of the membrane NADPH oxidase system [23]. The kidney expresses all main components of NADPH oxidase [24]. In this study, we provide a more complete picture of early events underlying the development of oxidative stress in RAS.

We found that the development of renovascular hypertension in RAS mice was associated with a rapid induction of *Ncf1* and *Ncf2*, which encode the NADPH oxidase subunits p47phox and p67phox, respectively. *Ncf1* and *Ncf2* expression were increased at three days and remained highly elevated in RAS mice through 28 days. Increased expressions of *Ncf1* and *Ncf2* have been described in several models of hypertension. In spontaneously hypertensive rats, *Ncf1* and *Ncf2* are highly expressed in vessels, the macula densa, distal tubules, cortical collecting ducts, and medullary collecting ducts [24]. Increased renal expression of *Ncf1* has been reported in the Dahl salt-sensitive rat [25] as well as in spontaneously hypertensive rats [24]. In addition to hypertension, *Ncf1* plays an essential role in the development of diabetic nephropathy in the Akita mouse [26].

*Nox4* is the renal homologue of the neutrophil NADPH oxidase subunit gp91phox and is expressed in proximal tubules and medullary collecting ducts [27]. We found that *Nox4* expression was decreased in RAS mice from 7–28 days following surgery. In accord with our observations, decreased *Nox4* expression has been reported in Ang II infused rats, which develop hypertension in association with increased oxidative stress [28]. In vascular smooth muscle cells, Ang II decreases *Nox4* expression [29]. There is some evidence indicating that *Nox4* may decrease chronic renal injury. Renal oxidative stress and fibrosis are significantly increased in *Nox4* deficient mice subjected to unilateral urinary obstruction [30], indicating that *Nox4* may have a protective role in at least some renal injury models [31].

Superoxide generated through action of NDPH oxidase is catabolized through action of superoxide dismutase (SOD). We detected three isoforms of SOD in renal cortex-the copper-zinc (Cu-Zn) SOD (*Sod1*), the mitochondrial manganese (Mn) SOD (*Sod2*), and the extracellular SOD (*Sod3*). We found that *Sod1* and *Sod2*, but not *Sod3* were slightly increased at three days following RAS surgery, but levels of all three SOD isoforms were decreased at 7, 14, and 28 days following surgery. Expression of *Ccs*, the copper chaperone that activates *Sod1* was not altered in RAS mice.

Our observation that increased DHE staining, indicative of superoxide generation, was associated with increased expression of NADPH oxidase subunits (*Ncf1* and *Ncf2*) and decreased expression of SOD isoforms is similar to observations made in other chronic renal disease models, including subtotal nephrectomy [32] and renovascular hypertension [33,34] and spontaneously hypertensive rats [35]. In rats infused with Ang II, the development of hypertension was associated with up regulation of NADPH oxidase and suppression of superoxide dismutase [36].

Although this is the first murine renal artery stenosis study to focus on early time points—within one week—on the development of renal atrophy, there are several limitations. Our approach was to employ PCR based array analysis to assess expression of a large number of redox-sensitive genes. We have not systematically assessed function of these genes, many of which are regulated through post-transcriptional mechanisms--for example, membrane translocation and assembly in the case of NAD(P)H oxidase subunits. However, other studies have shown that changes in NADPH oxidase subunit mRNA expression and protein production agree relatively well in spontaneously hypertensive rats [24]. We have not yet embarked on any therapeutic interventions—pharmacologic or genetic—to define the mechanistic relevance of the changes in gene expression that we have identified.

Our current study, which focuses on early events following initiation of RAS, has provided evidence for intra-renal activation of the renin angiotensin system, prior to increases in plasma renin activity. We provide evidence that renal parenchymal cells, rather than infiltrating inflammatory cells, play a major role in induction of chemokine generation early in the development of RAS and such induction occurs in the absence of any discernible morphologic alteration in the kidney at such early time points. We demonstrate that the production of reactive oxygen species occurs early in the development of RAS, and is associated with rapid induction of the NADPH oxidase components *Ncf1* and *Ncf2*. SOD isoforms expression are transiently increased, but decreases thereafter. A key issue that remains to be addressed relates to the point at which renal damage in RAS becomes irreversible, and whether it is possible to develop morphologic or biochemical biomarkers that predict this process. Our detailed observation of early time points described here may provide the basis for such studies.

## 4. Experimental Section

### 4.1. Animal Models

C57BL/KS male mice, 5–6 weeks old, were obtained from Jackson Laboratory (Bar Harbor, ME, USA). RAS surgery was performed as previously described [10–12] at 6–7 weeks of age (*N* = 10 for each time points). In brief, mice were anesthetized with isoflurane, a 0.5 mm length of 0.36 mm (OD) × 0.2 mm (ID) polytetrafluoroethylene tubing (Braintree Scientific, Braintree, MA, USA) was surgically placed around the right renal artery and held in place with two 10-0 nylon circumferential sutures (Surgical Specialties, Reading, PA, USA). Sham surgeries (*N* = 5 for each time points) consisted of a flank incision and mobilization of the renal artery without placement of a cuff.

Blood pressures were measured on conscious acclimatized mice using tail cuff method (CODA System, Kent Scientific, Torrington, CT, USA) at three days prior to surgery and at 3, 7, 14 and 28 days post-operative prior to sacrifice. Mice were euthanized by exsanguination at 3, 7, 14, and 28 days post-surgery. Kidneys and hearts were perfused with sterile PBS, excised, photographed, weighed and were either preserved immediately for histology or flash frozen in liquid nitrogen for Western blotting and PCR analysis. All animal protocols were approved by the Mayo Clinic Institutional Animal Care and Use Committee.

### 4.2. Fractionation of Renal Parenchymal Cells

A preparation of single cell suspension of renal cells were obtained as previously described [37] from kidneys of mice with RAS (*N* = 5 for each time points) and sham surgery (*N* = 5 for each time points) at three days and seven days post-surgery. In brief, renal cortex were minced, digested with collagenase (Collagenase I CLS4, Worthington, WA, USA) and DNase I (Roche, Indianapolis, IN, USA), and filtered through 70 μm mesh. The resulting suspension was incubated with CD11b antibody-conjugated magnetic micro beads and ran through VarioMACS System (Miltenyi Biotec, Auburn, CA, USA) to obtain the CD11b+ fraction. The same procedure is repeated with CD90 antibody-conjugated magnetic micro beads to obtain the CD90+ fraction and the CD11b−/CD90− fraction.

### 4.3. Biochemical Analysis

Blood was collected by tail bleed for serial measurements and by terminal bleed through the IVC. The plasma fraction was separated by centrifugation upon collection and stored at −80 °C until assay. Renin activity in plasma was assessed via production of angiotensin I from angiotensinogen using a commercially available GammaCoat Plasma Renin Activity 125I RIA kit (DiaSorin, Stillwater, MN, USA). Porcine angiotensinogen (A2283; Sigma-Aldrich, St. Louis, MO, USA) substrate was used for the assay.

### 4.4. Micro CT Imaging

Micro CT studies were done as previously described [38] on kidneys of 129S mice with RAS (*n* = 3) and sham surgery (*n* = 3) at 20 days post-surgery. In brief, mice were euthanized by exsanguination. Kidneys are perfused with heparinized saline, followed by intravascular contrast agent (0.8 mL/min) (Microfil MV122, Flow Tech, Carver, MA, USA), until the kidneys had a uniform yellow coloration. The kidneys were removed, immersed in 10% formalin, and embedded in a synthetic resin for Micro-CT scanning at 0.5° increments and analyzed with the Analyze™ software package (Analyzer 10.0, Biomedical Imaging Resource, Mayo Clinic, Rochester, MN, USA). Microvessel density was calculated as the total volume of vessels with diameter <0.52 mm normalized to the kidney volume.

### 4.5. Histology and Immunohistochemistry

Kidneys were fixed with 10% neutral buffered formalin and processed for histology or immunostaining using standard techniques. Histological section (5 μm thick) were prepared and stained with hematoxylin-eosin (H & E), anti-CD3 (DAKO, Carpinteria, CA, USA), and anti F4/80 (Abd Serotec, Raleigh, NC, USA). Atrophy was assessed semi quantitatively by measuring percentage area of atrophic tubules over the whole cortical surface. Immune cells infiltration was assessed by measuring percentage area with positive staining over the whole cortical surface, avoiding the perivascular regions of large vessels and medullary portions of the kidney. All measurements and quantification were performed in a random blinded fashion using a Leica DMLB microscope (Leica Microsystems, Buffalo Grove, IL, USA), a Micropublisher 3.3 RTV camera (QImaging, Surrey, BC, Canada), and the MetaVue Imaging System (V.6.3r2, Universal Imaging, Downington, PA, USA). Extent of oxidative stress was assessed by intensity of DHE staining (Sigma-Aldrich, St. Louis, MO, USA) on 4 μm frozen tissue section. Slides were then observed under identical exposure conditions to determine changes in staining intensity.

### 4.6. PCR Array

Kidneys for PCR Array studies were obtained from mice with RAS (*N* = 3 for each time points) and sham surgery (*N* = 3 for each time points) at three days and seven days post-surgery. Total RNA was extracted with RNeasy Mini Plus kit (Qiagen, Valencia, CA, USA). RNA quality was assessed using the Agilent2100 Bioanalyzer (Santa Clara, CA, USA) and reversed transcribed using the RT^2^ First Strand kit (SA Biosciences, Frederick, MD, USA). PCR were performed to evaluate expression of 84 oxidative stress related genes using RT^2^ profiler PCR array PAMM-065Z (Mouse Oxidative Stress and Antioxidant Defense) on the CFX96 (BioRad, Hercules, CA, USA). Analysis for relative changes in gene expression and heatmap was performed with SA Biosciences RT^2^ Profiler PCR Array Data Analysis software v3.5 using the comparative threshold cycle (ΔΔ*Ct*) method. Significant induction was determined for genes showing greater than two fold increase relative to sham with *p* value < 0.05.

### 4.7. mRNA Analysis

Total RNA was extracted with RNeasy Mini Plus kit (Qiagen, Valencia, CA, USA) and reversed transcribed using iScript cDNA synthesis kit (Bio-Rad, Hercules, CA, USA). Gene expression analysis was determined by quantitative real-time PCR using ABI 7900HT (Applied Biosystem Technology, Foster City, CA, USA) and normalized to 18s. The following primers were used: *18s* forward 5′-CTC AAC ACG GGA AAC CTC AC-3′; *18s* reverse 5′-CGC TCC ACC AAC TAA GAA CG-3′; *Ren1* forward 5′-GAG GTA GCG ACC CGC AGC ATT AT-3′; *Ren1* reverse 5′-GCG CTG CCT CCC AGG TCA AA-3′; *Agt* forward 5′-CGT GCG TGC CCC TAG GTG AG-3′; *Agt* reverse 5′-GGC AGA GTC AGG CGG ATG GC-3′; *Nox*4 forward 5′-AGG CTC CAG GCA AAC ACT GGG-3′; *Nox*4 reverse 5′-TGC AGC GAG GCA GGA GAG TCA-3′; *Gpx*6 forward 5′-CCA GAA GTT GTG GGG TTC CTG TC-3′; *Gpx*6 reverse 5′-GAC GGT GCC AGT CAC CCC TTT-3′; *Tnf*α forward 5′-GGG ACA AGG CTG CCC CGA CT-3′; *Tnf*α reverse 5′-TCC TTG GGG CAG GGG CTC TT-3′. All other primers were obtained from SA Biosciences (Frederick, MD, USA).

### 4.8. Statistical Analysis

Data are presented as means ± SE. On the basis of previous work in our laboratory, at least 5 mice in each group were required to detect a difference between groups (80% power and 5% type I error). Pair wise comparisons were done using student *t*-test for parametric data and Mann-Whitney test for non-parametric data or data without normal distribution. To assess differences between groups, ANOVA or a χ^2^ test was used when appropriate. A Bonferroni adjustment was used for posthoc comparison of the measurements. *p* values <0.05 were considered significant. Statistical analyses were performed with InStat version 3.00 (GraphPad Software, San Diego, CA, USA).

## 5. Conclusions

In this study, we provide evidence indicating that oxidative stress derives from renal parenchymal cells and provide the initial signals directing the subsequent influx of inflammatory cells to the kidney. Of note, these signals occur prior to the development of any significant histologic alterations or elevation of plasma renin activity. Further studies will be directed towards identification of redox sensitive chemokines generated by renal parenchymal cells during the development of renovascular hypertension.
